# Machine learning to characterize bone biomarkers profile in rheumatoid arthritis

**DOI:** 10.3389/fimmu.2023.1291727

**Published:** 2023-11-09

**Authors:** Giovanni Adami, Angelo Fassio, Maurizio Rossini, Camilla Benini, Riccardo Bixio, Denise Rotta, Ombretta Viapiana, Davide Gatti

**Affiliations:** Rheumatology Unit, University of Verona, Verona, Italy

**Keywords:** bone metabolism, bone markers, rheumatoid arthritis, bone mineral density (BMD), inflammation

## Abstract

**Background:**

Bone metabolism is disrupted in rheumatoid arthritis (RA); however, the bone metabolic signature of RA is poorly known. The objective of the study is to further characterize the bone metabolic profile of RA and compare it to psoriatic arthritis (PsA), systemic sclerosis (SSc) and healthy controls.

**Methods:**

We did a cross-sectional case-control study on consecutively enrolled patients and age-matched controls. We collected clinical characteristics, serum biomarkers related to bone metabolism and Bone Mineral Density (BMD). A multiple correlation analysis using Spearman's rank correlation coefficient was conducted within the RA patient group to investigate associations between biomarker levels and clinical variables. Machine learning (ML) models and Principal Component Analysis (PCA) was performed to evaluate the ability of bone biomarker profiles to differentiate RA patients from controls.

**Results:**

We found significantly lower BMD in RA patients compared to PsA, and Systemic Sclerosis SSc groups. RA patients exhibited higher Dkk1, sclerostin and lower P1nP and B-ALP levels compared to controls. No significant differences in CTX levels were noted. Correlation analysis revealed associations between bone biomarkers and clinical variables. PCA and ML highlighted distinct biomarker patterns in RA which can effectively discriminated bone biomarkers profile in RA from controls.

**Conclusion:**

Our study helped uncover the distinct bone profile in RA, including changes in bone density and unique biomarker patterns. These findings enhance our comprehension of the intricate links between inflammation, bone dynamics, and RA activity, offering potential insights for diagnostic and therapeutic advancements in managing bone involvement in this challenging condition.

## Introduction

Rheumatoid arthritis (RA), a chronic autoimmune disorder, exerts a profound influence on skeletal health, extending beyond its well-documented effects on joint structures (1, 2). The intricate interplay between RA and osteoporosis is of substantial clinical significance, necessitating a comprehensive understanding of the underlying mechanisms. Within this context, the exploration of bone turnover markers and modulators (BTMs), encompassing a spectrum of biochemical entities reflective of bone formation and resorption dynamics, has emerged as pivotal in deciphering the intricate relationship between RA and osteoporosis. BTMs offer dynamic insights into the equilibrium between bone formation and resorption, shedding light on the intricate processes governing skeletal metabolism. These markers can be categorized into those reflecting bone formation and those indicative of bone resorption (3, 4). In the context of RA, where the chronic inflammatory milieu poses a multifaceted challenge to bone health, BTMs assume particular significance (5). The Wnt signaling pathway stands as a pivotal regulator of BTMs and plays a central role in the maintenance of bone homeostasis (6). In particular, two key antagonists within this pathway, sclerostin and Dickkopf-1 (Dkk1), have garnered substantial scientific attention (7). The upregulation of sclerostin and Dkk1 is thought to hinder bone formation, thereby exacerbating the risk of osteoporosis and fragility fractures in RA patients. The mechanistic insights into the interplay between sclerostin and RA pathogenesis continue to be a subject of active research, with implications for therapeutic targeting.

Psoriatic arthritis (PsA) primarily characterized by bone apposition (8), and systemic sclerosis (SSc), a non-inflammatory disease known to cause osteoporosis (9), offer distinct models for understanding osteoporosis etiology in autoimmune diseases. The main goal of this study is to examine RA's biomarker profile, comparing it with healthy controls and two other representative autoimmune diseases.

## Materials and methods

### Study population

We conducted a cross-sectional study involving a total of 1883 consecutively enrolled participants, which included 1462 patients diagnosed with Rheumatoid Arthritis (RA), 60 patients with Psoriatic Arthritis (PsA), 62 patients with Systemic Sclerosis (SSc), and 359 age-matched healthy controls. All participants were recruited from the Rheumatology Section of the University of Verona Hospital between January 2012 and December 2019. The study was conducted according to the protocol REUMABANK approved by the University of Verona local Ethic Committee, in accordance with the 1964 Helsinki declaration and its later amendments or comparable ethical standards, and all participants provided informed consent.

### Data collection

For the RA patient group, we collected extensive clinical data at the time of serum sample collection, including disease duration, Disease Activity Score 28-C-Reactive Protein (DAS28-CRP), C-Reactive Protein (CRP) levels, Erythrocyte Sedimentation Rate (ESR) levels, Health Assessment Questionnaire (HAQ) scores, glucocorticoid (GC) cumulative dose, GC ongoing dose and disease modifying anti-rheumatic drugs (DMARDs).

### Serum biomarker analysis

Blood samples were drawn at morning fasting. Serum samples were aliquoted and stored at −80°C until they were assayed for C-terminal telopeptide of type I collagen (CTX, a marker of bone resorption), Procollagen I Intact N-Terminal Peptide (P1NP, a marker of bone formation), Bone Alkaline Phosphatase (B-ALP, a marker of bone formation), Dkk1 (a Wnt inhibitor), Sclerostin (a Wnt inhibitor), 25OH-Vitamin D (25OHVitD), Parathyroid hormone (PTH). CTX, P1nP and B-ALP were measured by the IDS-ISYS Multi-Discipline Automated Analyzer (Immunodiagnostic System, Boldon, UK) based on chemiluminescence technology. The coefficients of variation (CV) intra-assay measured were 3.0 % for P1NP, 2 % for CTX, and 4 % for B-ALP. Serum Dkk1 and sclerostin were measured by ELISA (Biomedica Medizinprodukte GmbH & Co KG, Wien, Austria) with sensitivities of 0.89 and 8.9 pmol/L and intra-assay coefficients of variation of 7.8 and 5.6 %, respectively. Inter-assay variabilities were 8.2 and 6.9 %, for Dkk1 and sclerostin, respectively. PTH was measured by ELISA (IDS Ltd. Boldon, UK) with intra-assay variability of 6 % and inter-assay variability of 7%. 25OHVitD was measured with LIAISON® 25OHVitD assay (DiaSorin, Italy), the intra-assay variability was 8% and the inter-assay variability was 12%. All samples were measured in a single batch in order to limit inter-assay variability.

### Bone mineral density measurement

BMD was measured at femoral neck and lumbar spine (L1–L4) by dual-energy X-ray absorptiometry (DEXA) (QDR Hologic Delphi) in patients with RA, PsA and SSc. BMD levels were not available in healthy controls. The variation coefficient was 1 % for vertebral site and 1.2 % for femoral neck.

### Group comparisons

To assess differences in biomarker levels and BMD among the study groups, we performed analysis of covariance (ANCOVA) adjusting for the following co-variates (cumulative glucocorticoid intake, age, gender, CRP levels, csDMARD and b/tsDMARD use) with Tukey's multiple comparisons *post-hoc* test.

### Correlation analysis

A multiple correlation analysis using Spearman's rank correlation coefficient was conducted within the RA patient group to investigate associations between biomarker levels and clinical variables. A heatmap was generated to visualize these correlations. ρ 0-0.19 indicates very weak correlation, 0.2-0.39 weak, 0.40-0.59 moderate, 0.6-0.79 strong and 0.8-1 very strong correlation. To account for multiplicity, we used the False Discovery Rate (FDR) approach with the two-stage step-up method of Benjamini, Krieger and Yekutieli (Q value 5% of FDR).

### Machine learning models

We employed machine learning (ML) techniques to evaluate the ability of biomarker profiles to differentiate RA patients from individuals with PsA and SSc. We ran three different ML models considering the following features in addition to serum biomarkers: cumulative glucocorticoid intake, age, gender, CRP levels, csDMARD and b/tsDMARD use: 1) Random Forest that ensemble learning method that combines multiple decision trees to make more accurate predictions. Each tree in the forest is constructed from a random subset of the data and a random subset of features. The final prediction is determined by aggregating the predictions of all individual trees. We optimized hyperparameters such as the number of trees (n=10), maximum depth (unlimited) to achieve the best classification performance. 2) Neural Network, which consist of interconnected layers of artificial neurons that process and transform data. In our case, a feedforward neural network with multiple hidden layers was employed. Hyperparameter optimization included tuning the number of hidden layers (n=100), regularization (α=0.001), maximal number of iterations (n=200). 3) Logistic Regression, which estimates the relationship between a set of independent variables (in this case, biomarker levels) and the probability of a specific outcome (RA, PsA, or SSc). We selected a Ridge (L2) regularization with strength C=1.

To assess the performance of these machine learning models, we employed standard evaluation metrics, including classification accuracy (CA), precision, recall, and receiver operating characteristic (ROC) curves with area under the curve (AUC) analysis. We implemented cross-validation (10 folds, stratified) to ensure the robustness of our models and mitigate overfitting.

### Principal component analysis

A Principal Component Analysis (PCA) was performed using all biomarkers to identify patterns and reduce dimensionality in the data. PCA was applied to reduce dimensionality of the dataset and find clusters of variables recording largely redundant information. PCs were selected based on eigenvalues explaining >75% of total variance.

### Missing values

Missing values (less than 2% of the overall dataset) were imputed using the most common value for that specific variable. We also conducted sensitivity analyses with complete dataset analyses (excluding subjects with missing values).

### Statistical analyses

Statistical analyses were performed using SPSS Version 26 (SPSS, Inc., Chicago, IL, USA) and GraphPad Prism version 9.5.1 (GraphPad Software, San Diego, CA, USA). The machine learning models were implemented using Orange: Data Mining Toolbox in Python Version 3.35.0. Hyperparameter tuning was conducted systematically to optimize each model's performance. PCA results were analyzed using PCA package on GraphPad Prism version 9.5.0 for Windows, GraphPad Software, San Diego, California USA.

## Results

### Characteristics of study participants

**Table 1** summarizes the demographic and clinical characteristics of the study participants. A total of 1883 participants were included in the analysis, comprising 1462 Rheumatoid Arthritis (RA) patients, 60 Psoriatic Arthritis (PsA) patients, 62 Systemic Sclerosis (SSc) patients, and 359 age-matched healthy controls. RA patients had a mean disease duration of 99 months, with an average Disease Activity Score 28-C-Reactive Protein (DAS28-CRP) of 2.8. The mean C-Reactive Protein (CRP) and Erythrocyte Sedimentation Rate (ESR) levels were 0.9 mg/L and 20 mm/h, respectively. The Health Assessment Questionnaire (HAQ) scores in RA patients averaged 0.750.

**Table 1:** Study population characteristics.

Characteristic	Rheumatoid Arthritis (RA) (n = 1462)	Psoriatic Arthritis (PsA) (n = 60)	Systemic Sclerosis (SSc) (n = 62)	Healthy Controls (n = 359)
**Age (years)**	58.9 ± 12.0	57.9 ± 12.3	63.9 ± 8.8	59.3 ± 15.6
**Gender (female/male)**	1241 / 221	42 / 18*	62 / 0*	192 / 167*
**Disease Duration (months)**	99 (48–178)	85 (35–165)	106 (68–200)	N/A
**DAS28-CRP**	2.8 ± 4.6	N/A	N/A	N/A
**CRP (mg/L)**	2.9 (0.3-5.0)	0.4 (0.1-1.2)	2.8 (0.7-3.8)	N/A
**ESR (mm/h)**	20 (12–30)	N/A	N/A	N/A
**HAQ Score**	0.750 (0.250-1.250)	N/A	N/A	N/A
**Median GC cumulative dose (g)**	11.5 (0-15.7)	0 (0-1.0)	0.3 (0-0.9)	N/A
**csDMARD (yes/no)**	1257 / 205	24 / 36*	19 / 43*	N/A
**b/tsDMARD (yes/no)**	603 / 859	30 / 30	2 / 60*	N/A

Values are presented as mean ± standard deviation unless otherwise specified. DAS28-CRP, Disease Activity Score 28-C-Reactive Protein; CRP, C-Reactive Protein; ESR, Erythrocyte Sedimentation Rate; HAQ, Health Assessment Questionnaire; GC, Glucocorticoid. * p<0.05 vs RA.

### Bone mineral density

**Figure 1:**
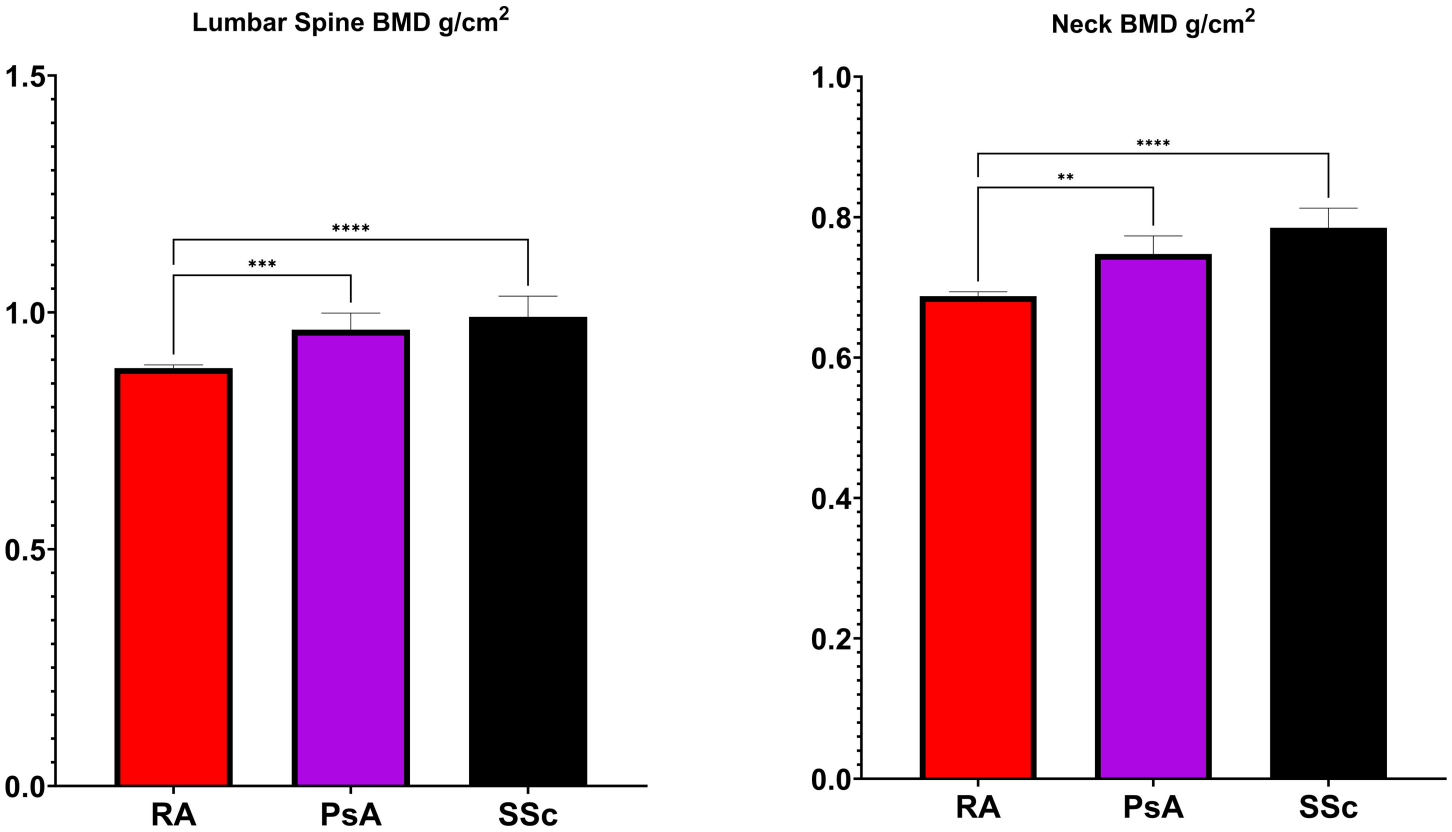
Bone Mineral Density (BMD) levels in rheumatoid arthritis (RA), psoriatic arthritis (PsA) and systemic sclerosis (SSc). *p <0.05; **p <0.01; ***p <0.001; ****p <0.0001.

**Figure 1** displays the results of BMD measurements at the femoral neck and lumbar spine. The BMD at the femoral neck was significantly lower in RA (0.687 ± 0.118 g/cm²) compared to PsA (0.747 ± 0.099 g/cm², p <0.001) and SSc (0.785 ± 0.118 g/cm² p<0.0001). Similarly, BMD at the lumbar spine was reduced in RA (0.882 ± 0.133 g/cm²) compared to PsA (0.963 ± 0.136 g/cm², p<0.01) and SSc (0.990 ± 0.173 g/cm² p<0.0001).

### Biomarker levels comparison

**Figure 2:**
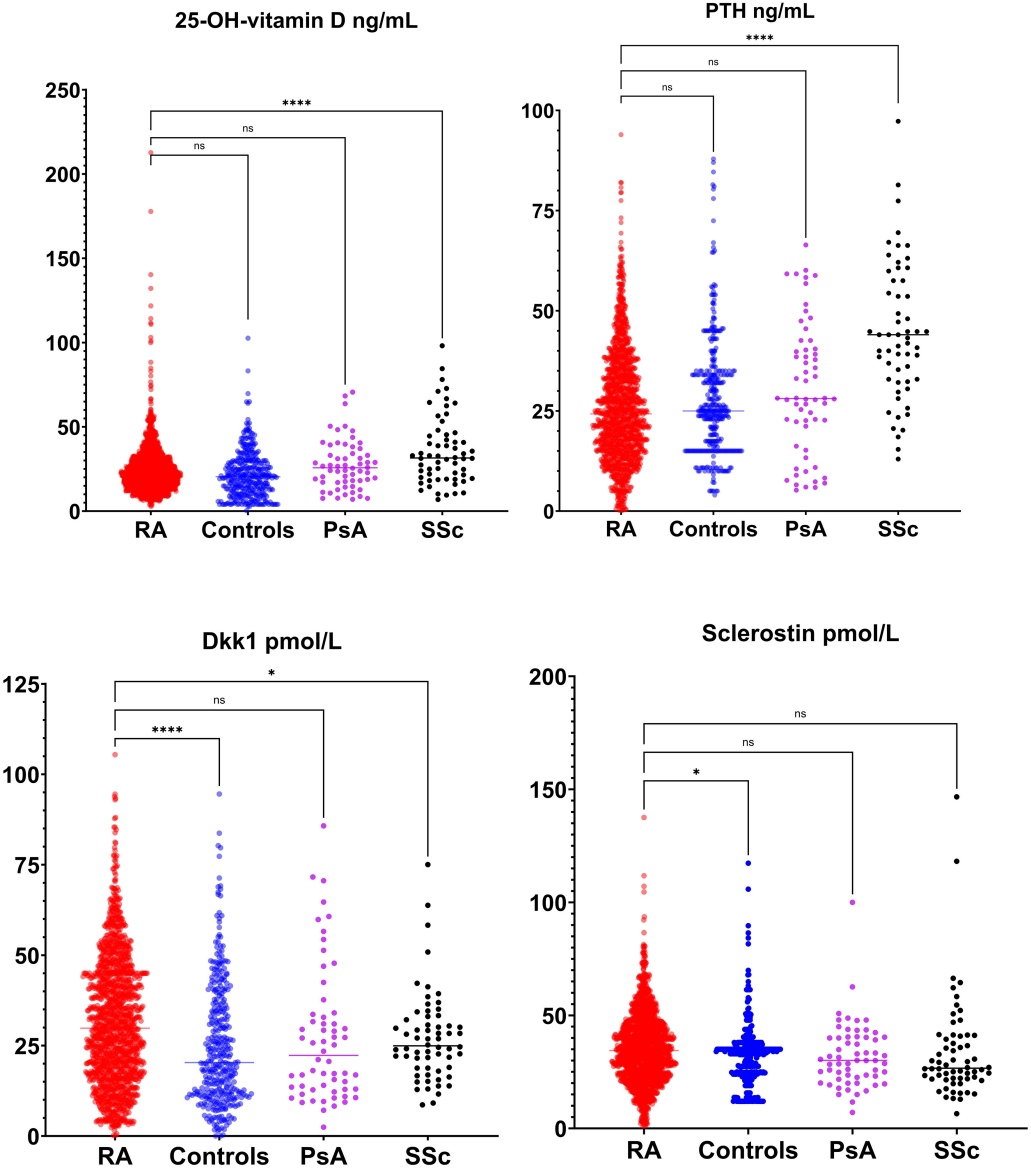
Bone modulators levels (25-OH-vitamin D, parathyroid hormone [PTH], dickopf 1 [Dkk1] and sclerostin) in rheumatoid arthritis (RA), healthy control, psoriatic arthritis (PsA) and systemic sclerosis (SSc). *p <0.05; **p <0.01; ***p <0.001; ****p <0.0001.

**Figure 3:**
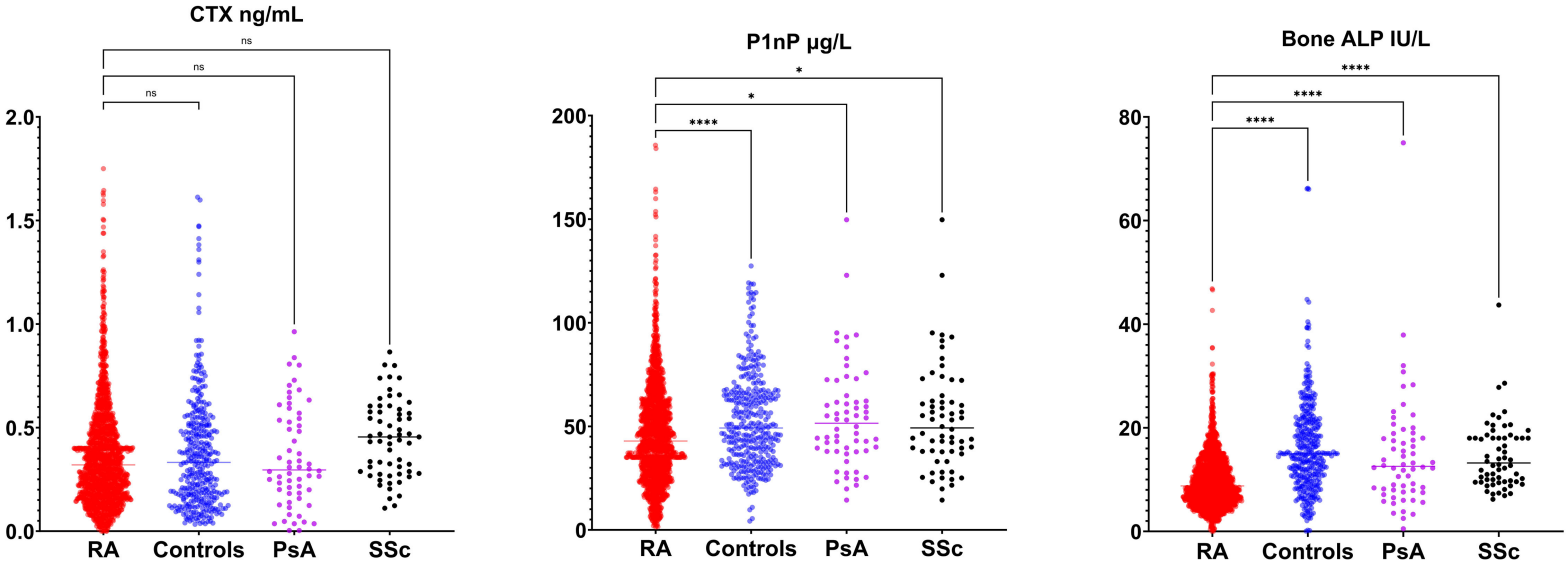
Bone turnover markers (C-terminal telopeptide [CTX], amino-terminal propeptide of type 1 procollagen [P1nP], bone alkaline phosphatase [ALP]) in rheumatoid arthritis (RA), healthy control, psoriatic arthritis (PsA) and systemic sclerosis (SSc). *p <0.05; **p <0.01; ***p <0.001; ****p <0.0001.

**Figures 2** and **3** present the results of the analysis comparing biomarker levels among the different study groups P1nP levels in the RA group (46.6 ± 25.2 µg/L) were significantly lower than those in healthy control (52.9 ± 23.0 µg/L, p<0.001) groups, PsA (54.4 ± 25.3 µg/L p<0.01) and SSc (53.6 ± 25.3 µg/L p<0.01). Bone ALP levels were lower in RA patients (9.9 ± 5.4 U/L) compared to healthy controls (16.3 ± 9.1 U/L, p<0.001), PsA (14.8 ± 11.1 U/mL, p<0.0001) and SSc (14.6 ± 6.5 U/mL, p<0.001). CTX levels did not differ between groups. Sclerostin levels were increased in RA patients (35.5 ± 15.6 pmol/mL) compared to healthy controls (32.8 ± 13). Dkk1 levels were significantly higher in the RA group (31.8 ± 17.4 pmol/L) compared to healthy controls (25.1 ± 17.2 pmol/L p<0.0001) and SSc (27.2 ± 12.4 pmol/L p<0.01) but not to PsA (27.3 ± 18.8 pmol/L, p 0,051) but were not different in PsA and SSc. PTH levels in RA patients (26.6 ± 16.2 ng/mL) were significantly lower than those in SSc (46.8 ± 20.6 ng/mL, p<0.0001) but did not differ from healthy controls and PsA. 25-OH-vitamin D serum levels were significantly higher in SSc compared to other groups but did not differ between RA and PsA or healthy controls. We did not have access to data on anti-resorptive treatment for the overall cohort. To control for the confounding introduced by anti-resorptive we have conducted a sub-analysis on patients with CTX >0.2 ng/mL (in whom bone turnover is not suppressed and active treatment with anti-resorptive is unlikely). Sub analysis on these patients is presented in **Supplementary Material Figure 1**. CTX was significantly higher in RA compared to healthy controls, P1nP and B-ALP lower in RA compared to healthy controls, BMD was lower in RA compared to PsA and SSc. Sensitivity analyses with complete dataset (excluding subjects with missing values) yielded similar results (data not shown).

### Correlation analysis

**Figure 4:**
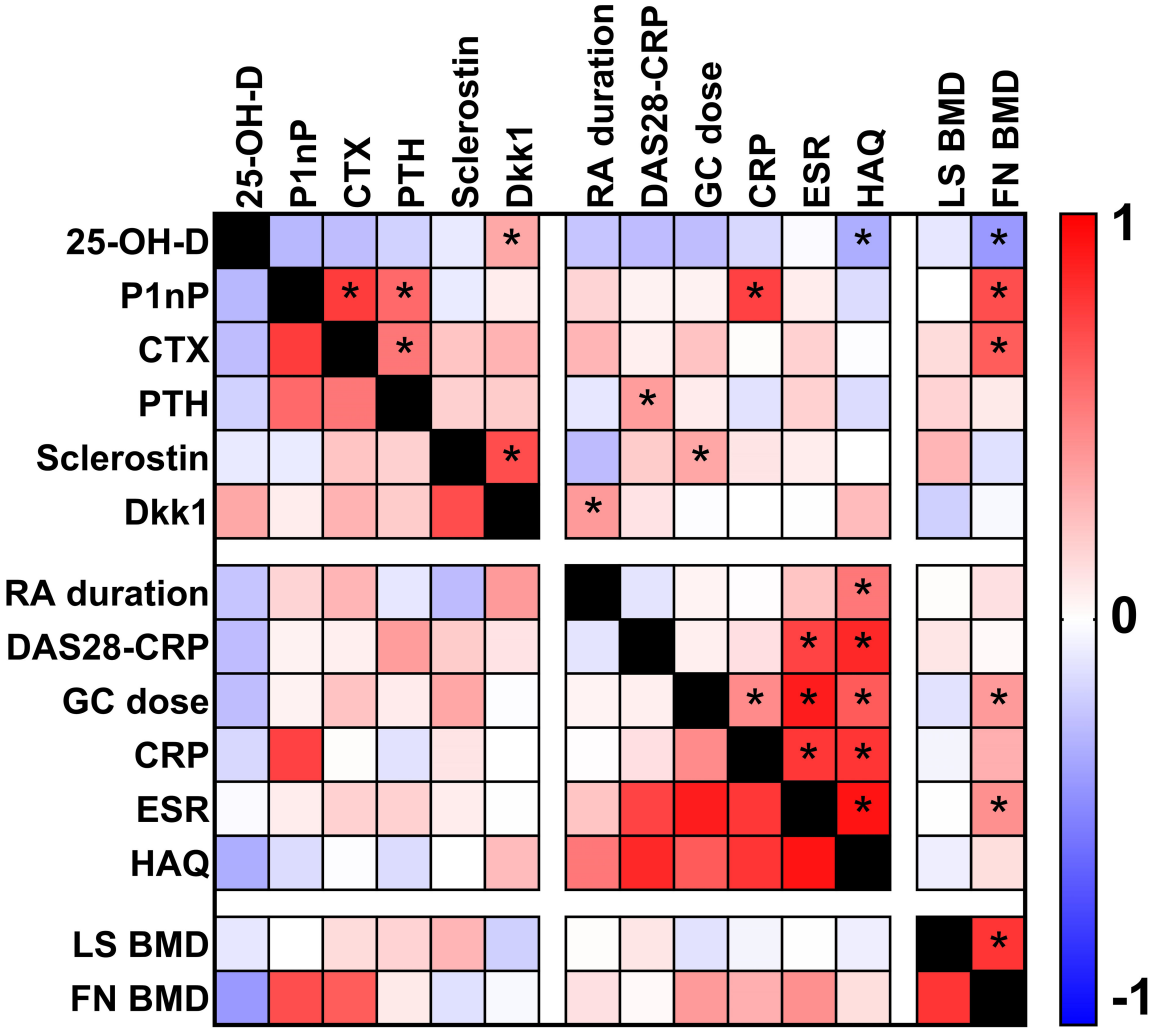
Heat map plot showing the correlations (Spearman's rank correlation analysis) between biomarkers and clinical parameters in patients with rheumatoid arthritis. Asterisks show p value <0.05.

In RA patients, Spearman's rank correlation analysis (**Figure 4**) revealed several significant associations between biomarkers and clinical variables. In particular, 25-OH-vitamin D was negatively associated with HAQ levels (ρ -0.131, p value 0.038), P1nP serum levels were positively associated with CRP levels (ρ 0.320, p value <0.001), PTH serum levels were positively associated with DAS28-CRP score (ρ 0.134, p value 0.034), sclerostin serum levels were positively associated with GC cumulative dose (ρ 0.145, p value 0.029) and Dkk1 serum levels were positively associated with RA disease duration (ρ 0.129, p value 0.045).

### Principal component analysis

**Figure 5:**
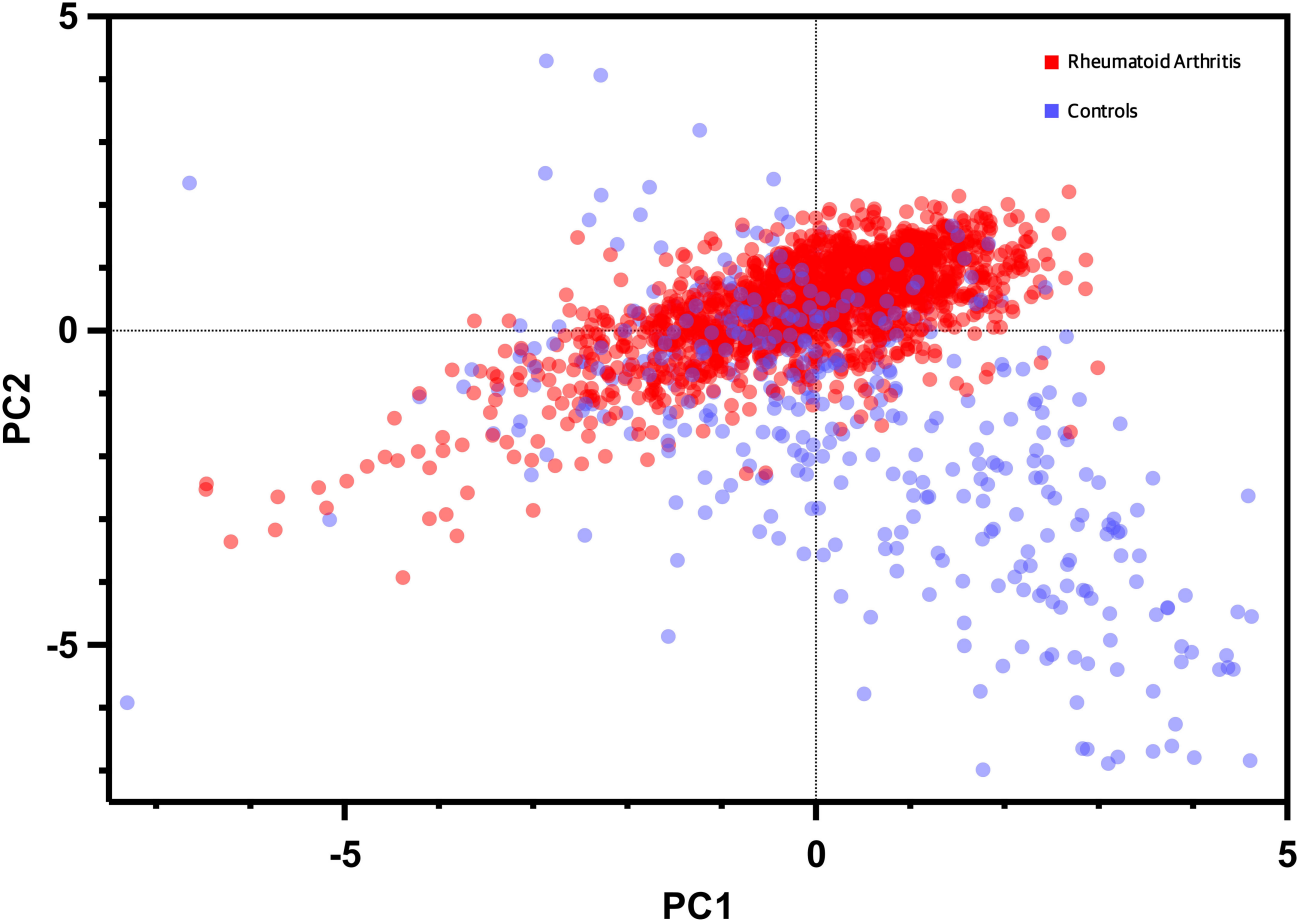
Dot plot of principal component analysis (PCA) based on biomarkers expression and bone mineral density levels. Each data point represents an individual participant, with the x-axis denoting the first principal component (PC1) and the y-axis representing the second principal component (PC2). The distribution of data points reflects the multidimensional clustering patterns based on the combined information of the biomarker, BMD, and clinical variables enabling visualization of distinctive subgroups and relationships within the study population.

The Principal Component Analysis (PCA) results are illustrated in **Figure 5**. This PCA plot demonstrates the distribution of study participants in the multidimensional space defined by the biomarkers. The clustering patterns and relationships among participants are evident, providing valuable insights into the differentiation of RA from other rheumatic diseases and healthy controls. The x-axis of the PC Score graph represents the first principal component (PC1), which is the most influential linear combination of the biomarkers. The y-axis, corresponding to the second principal component (PC2), captures the second-largest source of variation orthogonal to PC1. Each data point on the PC Score graph corresponds to an individual study participant, with their position determined by their biomarker profile's projection onto the PC1 and PC2 axes. Data points that cluster closely together on the graph share similar biomarker profiles. This clustering reflects the presence of distinct subgroups within our study population, characterized by specific patterns of biomarker expression. The PC Score graph allows us to explore whether the biomarker profiles of patients with RA form distinct clusters compared to controls. Moreover, it aids in identifying any potential overlap or separation between disease groups, shedding light on the discriminative power of these markers.

### Machine learning models

**Figure 6:**
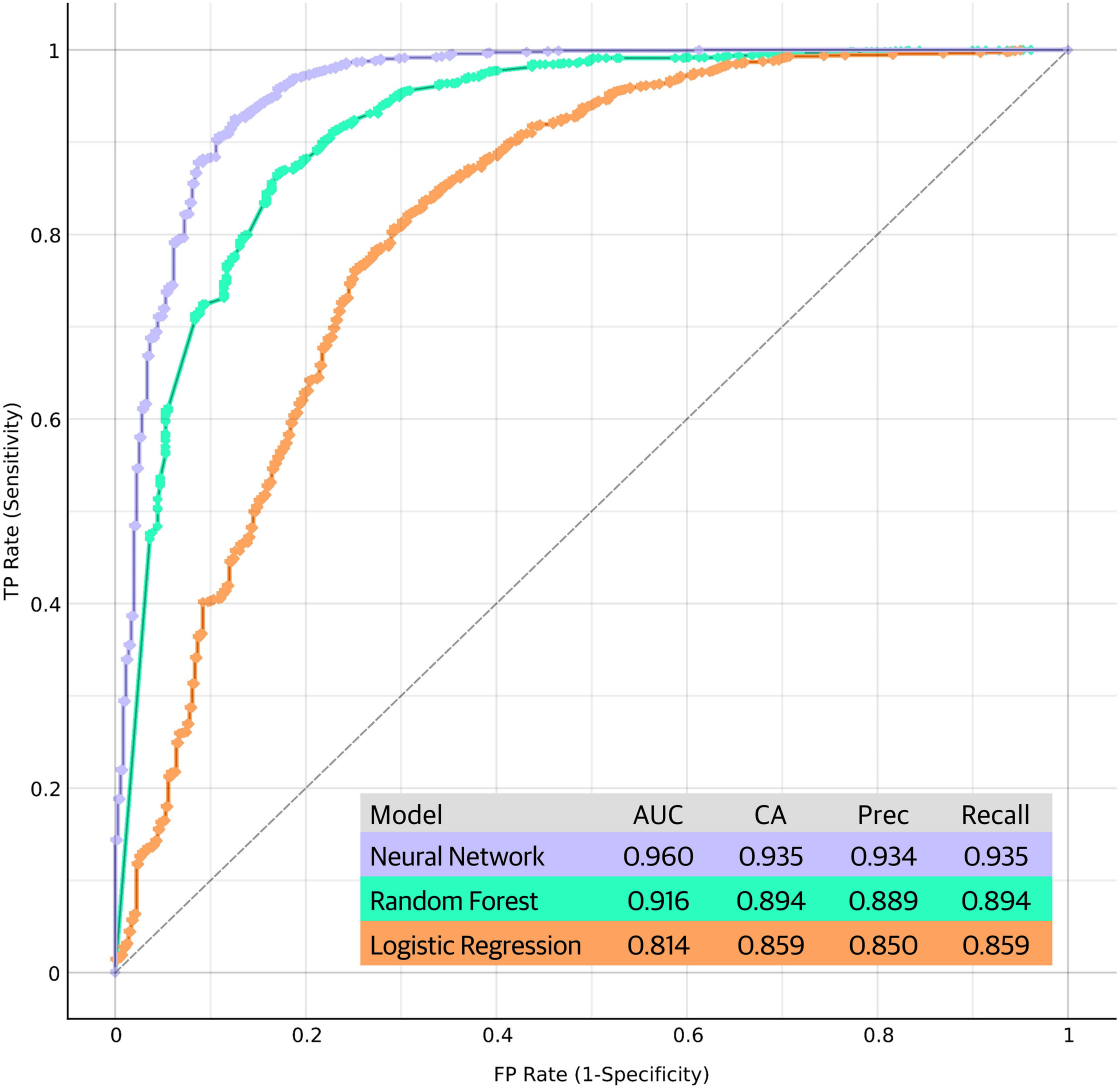
Performance metrics and receiver operating characteristic (ROC) curves of the machine learning models in discriminating RA patients from controls based on their biomarker profiles. The ROC curves plot the true positive rate (sensitivity) against the false positive rate (1-specificity) at various threshold settings, providing a comprehensive visualization of the models' discriminative power. Each curve represents the model's performance in distinguishing RA cases from controls, with the area under the curve (AUC) serving as an indicator of the overall predictive accuracy. Performance of the models are shown in the figure. CA, classification accuracy; Prec, precision.

**Figure 6** summarizes the performance metrics of the machine learning models in discriminating RA patients from controls based on their biomarker profiles. The results indicate that ML models can effectively discriminate RA from age-matched controls and Neural Network model performed better than the other two models. More complete set of performance metrics is shown in **Supplementary Materials** (classification accuracy curve, calibration curve and F1 curve).

## Discussion

Herein, we aimed to comprehensively characterize BTMs, bone modulators and clinical parameters in a large cohort of RA patients and compare them with individuals with PsA, SSc, and age-matched healthy controls. Our findings shed light on the distinctive bone signature of RA and provide valuable insights into its pathophysiological mechanisms.

Osteoporosis is a key feature of RA, and it has been shown to be, at least in part, independent from GC use (10–12). Indeed, the pathogenesis of systemic and local bone loss in RA is complex and many metabolic pathways are likely to play a role. Among these mechanisms the Wnt system has emerged as a crucial regulator (7, 13–15). We found that Dkk1 levels were significantly higher in RA patients compared to healthy controls and SSc. Of note, we also found a trend toward difference with the PsA group (p=0.051). Similarly, sclerostin, another antagonist of the Wnt pathway, was elevated in RA patients compared to healthy controls, potentially contributing to the altered bone homeostasis observed. In line with these observations, P1nP and B-ALP levels were markedly reduced in RA patients compared to both healthy controls and PsA. The impaired bone formation might be central in RA-associated bone pathology, possibly due to the suppressive effects of low-grade chronic inflammation on osteoblastic activity (16, 17). Interestingly, CTX levels did not differ significantly among groups, this seemingly counterintuitive observation could potentially be attributed to the inclusion of patients with, on average, long-term disease duration and most of them experiencing a state of low disease activity. Moreover, further investigations into specific CTX subtypes and their association with RA disease activity may reveal additional insights. In aggregate we can speculate that, in long standing RA, bone loss is mainly driven by impaired bone formation which is governed by altered Wnt system.

Our correlation analysis in RA patients unveiled intriguing relationships between biomarkers and clinical variables. 25-OH-vitamin D displayed a negative association with HAQ scores, emphasizing the role of vitamin D in functional status and disease severity in RA which has been reported in other cohorts (18–21). However, this association cannot prove a causal relationship but at least support a role of 25-OH-vitamin D as a proxy of severe disease and worse outcomes. Positive associations between P1nP and CRP levels and between PTH and DAS28-CRP scores suggest intricate interactions between inflammation, bone turnover, and disease activity in RA. We previously showed that PTH levels are the most important single determinant of Dkk1 levels (14, 22, 23), and their simultaneous elevation might predispose to bone erosions (13, 14, 24). Nonetheless, inflammation levels of the RA cohort were low on average, and we cannot generalize the present results to patients with active disease and more prominent inflammation. Similarly, the positive association of sclerostin levels with glucocorticoid cumulative dose despite being thought-provoking from a pathophysiological point of view, might have been triggered by the long duration of the disease which is, not surprisingly, highly correlated with GC cumulative dose. Additionally, the positive association between Dkk1 levels and RA disease duration suggests that Wnt pathway dysregulation may be linked to disease progression. Notwithstanding that, romosozumab, a sclerostin inhibitor, yielded encouraging results in preventing and treating GC induced osteoporosis (GIOP) in RA patients corroborating our finding (25–28).

Our PCA analysis revealed distinct clustering patterns among the study participants, reflecting unique biomarker profiles associated with RA group. The PCA approach allowed us to visualize the multidimensional nature of the biomarker data, highlighting potential biomarker combinations that contribute to the differentiation of RA from other rheumatic diseases and healthy controls. This supports the notion that RA possesses a characteristic bone signature, distinct from other rheumatic conditions. Furthermore, the ML analysis demonstrated promising results in the discrimination of RA patients from age-matched healthy controls based on their biomarker profiles. The Neural Network model exhibited superior performance, suggesting that complex relationships among biomarkers may hold key information for disease classification. These findings suggest that ML approaches could serve as valuable tools for the development of diagnostic or prognostic biomarker panels in RA.

Our study has strengths and limitations. We employed a cross-sectional design, which limits our ability to establish causality or determine the temporal relationships between biomarkers, clinical parameters, and disease progression. RA is a highly heterogeneous disease with varying clinical manifestations and disease courses. In this study, we focused on a specific subset of RA patients with long-term disease duration and low disease activity. Consequently, the results may not fully capture the diversity of the RA population, particularly those with more active disease or shorter disease duration. Although we collected extensive clinical data, including disease duration, DAS28-CRP, CRP levels, ESR levels, HAQ scores, and GC dose, other relevant clinical variables, such as radiographic joint damage, were not included in our analysis. These factors can potentially influence biomarker levels and bone health in RA patients. While we aimed to age-match healthy controls to the RA patient group, other factors such as lifestyle, dietary habits, and comorbidities were not accounted for in the control group. These uncontrolled variables could introduce confounding effects on biomarker levels and BMD measurements. Biomarkers assessed in this study represent only a subset of the intricate pathways involved in bone remodeling. Additional bone-related biomarkers and factors may contribute to the overall understanding of bone metabolism in RA. The machine learning models used for disease classification were trained and tested on the same dataset. External validation on an independent cohort of RA patients and controls is essential to assess the generalizability and robustness of the models in real-world clinical settings.

In conclusion, our study has provided comprehensive insights into the bone signature of RA, characterized by alterations in bone density and distinctive biomarker profiles. These findings contribute to our understanding of the complex interplay between inflammation, bone turnover, and disease activity in RA, with potential implications for diagnostic and therapeutic strategies in the management of bone involvement in this debilitating disease.

## Data availability statement

The raw data supporting the conclusions of this article will be made available by the authors, without undue reservation.

## Ethics statement

The studies involving humans were approved by Ethic committee of the Verona Hospital protocol REUMABANK. The studies were conducted in accordance with the local legislation and institutional requirements. The participants provided their written informed consent to participate in this study.

## Author contributions 

GA: Conceptualization, Data curation, Formal analysis, Investigation, Methodology, Visualization, Writing – original draft, Writing – review & editing. AF: Investigation, Validation, Visualization, Writing – review & editing. MR: Investigation, Supervision, Validation, Visualization, Writing – review & editing. CB: Investigation, Validation, Visualization, Writing – review & editing. RB: Investigation, Validation, Visualization, Writing – review & editing. DR: Investigation, Validation, Visualization, Writing – review & editing. OV: Investigation, Validation, Visualization, Writing – review & editing. DG: Investigation, Supervision, Validation, Visualization, Writing – original draft.
