# Supplementary material for: Machine learning to characterize bone biomarkers profile in rheumatoid arthritis
Source: Front Immunol. 2023 Nov 9;14:1291727. doi: 10.3389/fimmu.2023.1291727 (PMC10665911; doi:10.3389/fimmu.2023.1291727)

**Supplementary materials**

Title: Machine learning to characterize bone biomarkers profile in rheumatoid arthritis

Authors: Giovanni Adami1, Angelo Fassio1, Maurizio Rossini1 Camilla Benini1, Riccardo Bixio1, Denise Rotta1, Ombretta Viapiana1, Davide Gatti1

1. Rheumatology Unit, University of Verona, Verona, Italy

**eFigure 1.** Bone Mineral Density (BMD) levels in rheumatoid arthritis (RA), psoriatic arthritis (PsA) and systemic sclerosis (SSc) with CTX serum levels >0.200 ng/mL


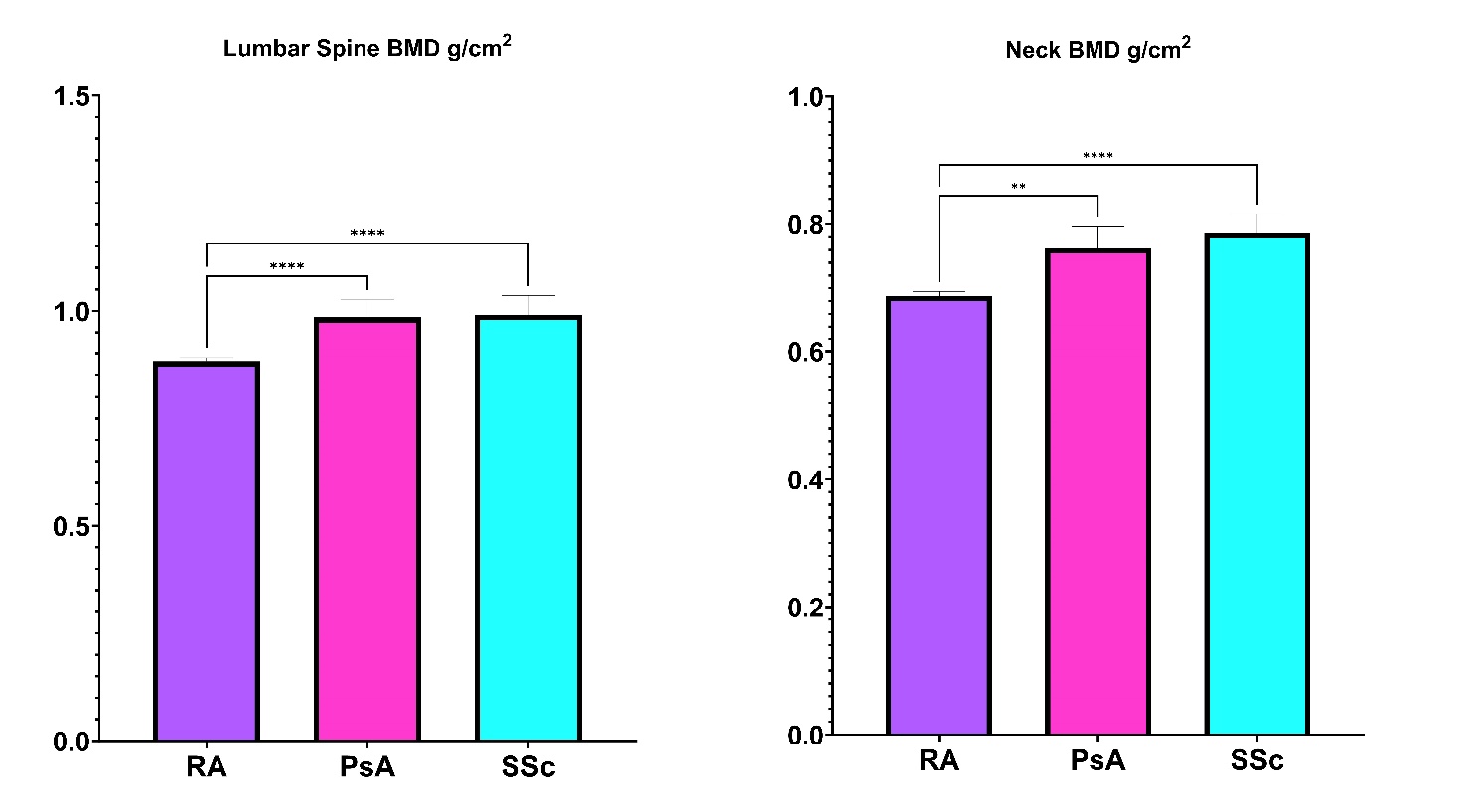


**eFigure 2.** Bone modulators levels (25-OH-vitamin D, parathyroid hormone [PTH], dickopf 1 [Dkk1] and sclerostin) in rheumatoid arthritis (RA), healthy control, psoriatic arthritis (PsA) and systemic sclerosis (SSc) with CTX serum levels >0.200 ng/mL


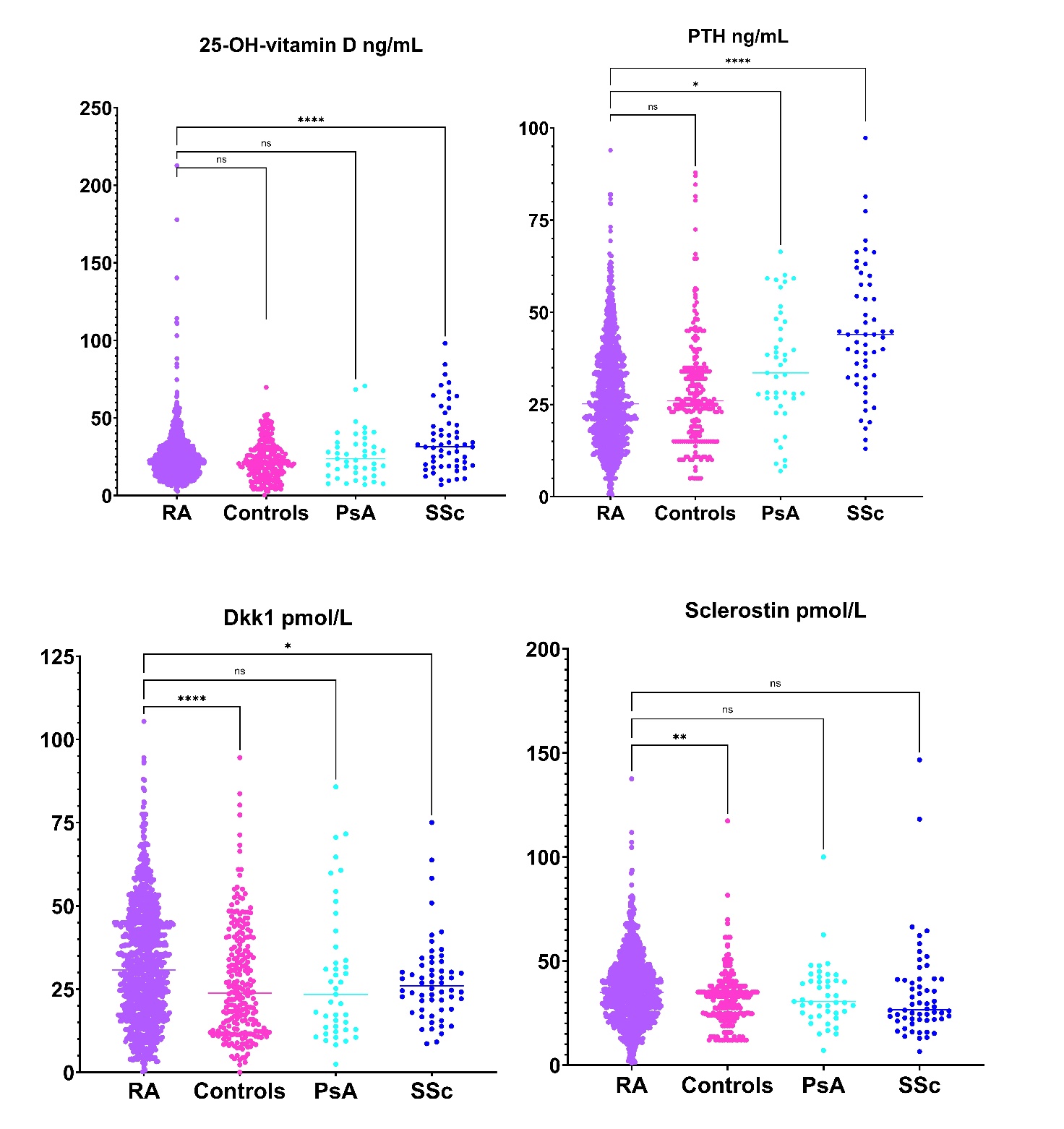


**eFigure 3.** Bone turnover markers (C-terminal telopeptide [CTX], amino-terminal propeptide of type 1 procollagen [P1nP], bone alkaline phosphatase [ALP]) in rheumatoid arthritis (RA), healthy control, psoriatic arthritis (PsA) and systemic sclerosis (SSc) with CTX serum levels >0.200 ng/mL


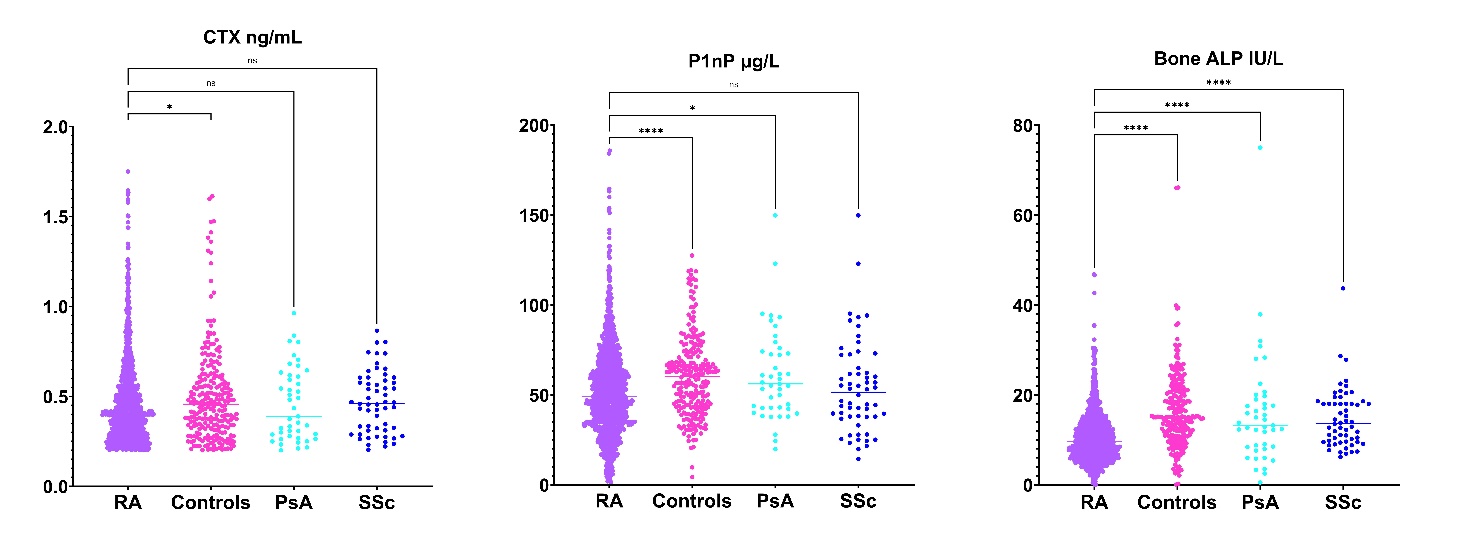


**eFigure 4.** Classification accuracy curve depicting the performance evaluation of the machine learning models, including Neural Network (blue), Random Forest (green), and Logistic Regression (orange), in discerning Rheumatoid Arthritis (RA) patients from controls based on their distinct biomarker profiles. The curve illustrates the classification accuracy, computed as the ratio of the correctly classified instances to the total instances, across different threshold values. Each point on the curve represents the model's accuracy at a specific threshold setting, providing a comprehensive overview of the models' discriminatory capacity.


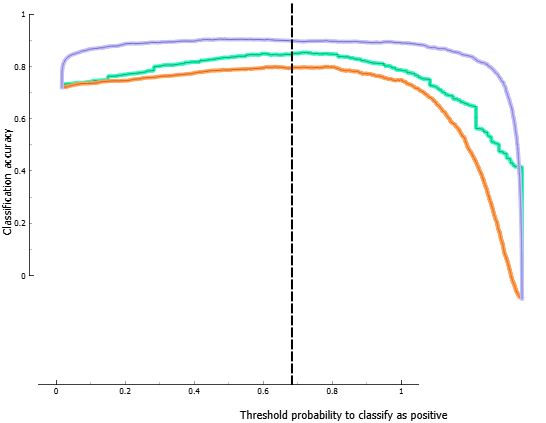


**eFigure 5.** Calibration curve illustrating the calibration performance of the machine learning models, including Neural Network (blue), Random Forest (green), and Logistic Regression (orange), in predicting Rheumatoid Arthritis (RA) based on their distinctive biomarker profiles. The curve demonstrates the relationship between predicted probabilities and the true fraction of RA cases, allowing an assessment of the models' calibration accuracy. Each point on the curve represents the agreement between the predicted and observed outcomes, providing valuable insights into the models' reliability and predictive validity.


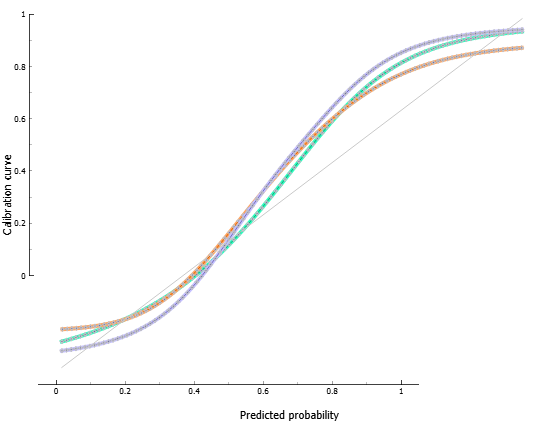


**eFigure 6.** F1 curve depicting the F1-score performance evaluation of the machine learning models, including Neural Network (blue), Random Forest (green), and Logistic Regression (orange), in discerning Rheumatoid Arthritis (RA) patients based on their distinctive biomarker profiles. The curve showcases the harmonic mean of precision and recall, providing a comprehensive assessment of the models' accuracy in capturing the true positives and minimizing false positives. Each point on the curve represents the F1-score at various threshold settings, highlighting the models' effectiveness in achieving a balance between precision and recall


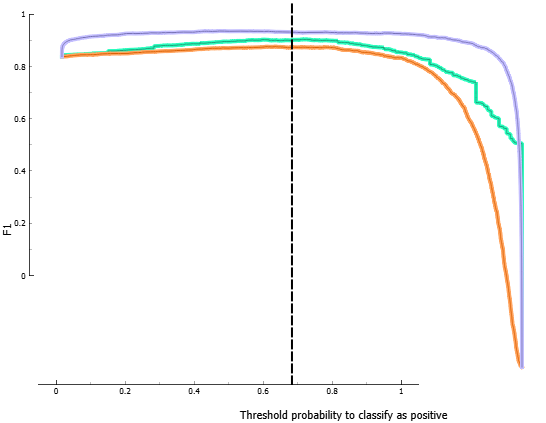

Supplement: Supplementary file 1 [file DataSheet_1.docx]
